# Influences of Deqi on Immediate Analgesia Effect of Needling SP6 (Sanyinjiao) in Patients with Primary Dysmenorrhea in Cold and Dampness Stagnation Pattern: Study Protocol for a Randomized Controlled Trial

**DOI:** 10.1155/2015/238790

**Published:** 2015-07-30

**Authors:** Yu-qi Liu, Peng Zhang, Jie-ping Xie, Liang-xiao Ma, Hong-wen Yuan, Jing Li, Chi Lin, Pei Wang, Guo-yan Yang, Jiang Zhu

**Affiliations:** ^1^School of Acupuncture Moxibustion and Tuina, Beijing University of Chinese Medicine, Beijing 100029, China; ^2^Institute of Basic Research in Clinical Medicine, China Academy of Chinese Medical Sciences, Beijing 100700, China; ^3^Beijing Tongren Hospital Affiliated to Capital Medical University, Beijing 100730, China; ^4^The Key Unit of State Administration of Traditional Chinese Medicine, Evaluation of Characteristic Acupuncture Therapy, Beijing 100029, China; ^5^School of Traditional Chinese Medicine, Capital Medical University, Beijing 100069, China

## Abstract

Deqi, according to traditional Chinese medicine, is a specific needle sensation during the retention of needles at certain acupoints and is considered to be necessary to produce therapeutic effects from acupuncture. Although some modern researches have showed that Deqi is essential for producing acupuncture analgesia and anesthesia, the data are not enough. It is a paper of a multicenter, randomized controlled study protocol, to evaluate the influences of Deqi on acupuncture SP6 in Cold and Dampness Stagnation pattern primary dysmenorrhea patients, in terms of reducing pain and anxiety, and to find out the relationship between Deqi and the temperature changes at SP6 (Sanyinjiao) and CV4 (Guanyuan). The results of this trial will be helpful to explain the role of Deqi in acupuncture analgesia and may provide a new objective index for measuring Deqi in the future study. This trial is registered with ChiCTR-TRC-13003086.

## 1. Introduction

Deqi, according to records in both ancient and modern books of traditional Chinese medicine (TCM), is a specific needle sensation which usually occurs during retention of acupuncture needles at certain acupoints, and both patients and acupuncturists can feel the Deqi sensation. It is regarded as a necessary factor for producing effects from acupuncture following traditional Chinese medicine theory. Patients can feel Deqi as multiple sensations at the needling acupoints and along the meridians sometime, such as soreness, numbness, distension, or a minimal muscular contraction around the needle [1–3] and also objective physiological changes, such as the skin temperature changes at the acupoints [4] or the response of brain [5, 6]. For acupuncturists, they can feel a change of the mechanical behavior of the tissues surrounding the needle, such as an increase of the force necessary to pull the needle out of the tissue (pullout force) [7]. Although some modern research showed that Deqi is essential for producing acupuncture analgesia and anesthesia [8], new evidence to confirm the conclusion is still urgently needed [9, 10].

Primary dysmenorrhea (PD) refers to painful menstrual cramps without any evident pathology. It is characterized by crampy suprapubic pain with radiation into the lower quadrants, the lumbar area, and the thighs [11]. PD is a common gynecological complaint and significantly affects study, work, sports, and social activities [12–18]. So far nonsteroidal anti-inflammatory drugs (NSAIDs) or oral contraceptive pills (OCPs) are widely advocated as standard treatments for women with PD [19, 20]. However, acupuncture as nonpharmacological approaches has great potential value.

Acupuncture is one of the main treatment modalities of TCM. Several trials [21, 22] have already demonstrated the encouraging results of acupuncture as a nonpharmacological option for the treatment of PD. Two systematic reviews also demonstrated the effect [23] and cost-effectiveness [24] of this therapy. The acupoint SP6 (Sanyinjiao) is found to be one of the most commonly used points encountered when searching ancient Chinese medical classics, Chinese acupuncture textbooks, and clinical trials using acupuncture-related therapies for PD [25, 26]. Previous randomized controlled trials have showed that acupuncture at SP6 (Sanyinjiao) can relieve the pain of PD immediately [27–29] especially for PD patients with Cold and Dampness Stagnation pattern [30], which is the most common pattern in PD patients [31, 32]. Langevin's study [7] also showed that, compared with the nonacupoint control group, the average pullout force in SP6 (Sanyinjiao) was greater, which suggested that SP6 (Sanyinjiao) might be especially sensitive in Deqi.

In this study, we will perform a randomized controlled trial using SP6 to investigate the influences of Deqi on immediate analgesia effects in PD patients with Cold and Dampness Stagnation pattern. The primary objective of this trial is to evaluate the influences of Deqi on acupuncture therapy, in terms of pain reduction (measured on a 0–100 mm visual analogue scale for pain (VAS-P)) achieved before and after intervention (i.e., before and after 30 minutes of treatment). The secondary objectives are as follows: (1) to evaluate the influences of anxiety reduction on a 0–100 mm visual analogue scale for anxiety (VAS-A) and (2) to evaluate the influences of temperature changes at SP6 (Sanyinjiao) and CV4 (Guanyuan) acupoints monitor by a digital infrared thermographic imaging device (only in the Dongzhimen Hospital).

## 2. Methods

### 2.1. Ethics

The trial protocol is in accordance with the principle of the Declaration of Helsinki [33] and has been approved by the Ethic Committee of Beijing University of Chinese Medicine (Beijing, China, Approval number 2012-040). Each participant will be notified regarding the study protocol. Written informed consent will be obtained from each participant.

### 2.2. Settings and Participants

A target sample of 96 participants will be recruited in acupuncture clinic from the following four hospitals: Dongzhimen Hospital Affiliated to Beijing University of Chinese Medicine, Beijing Hospital of Traditional Chinese Medicine affiliated to Capital Medical University, Huguosi Hospital of Traditional Chinese Medicine affiliated to Beijing University of Chinese Medicine, and Hebei Medical University. The trial will be conducted from March 2013 to March 2015.

#### 2.2.1. Sample Size

This is a study without similar references as we know and 40 patients per group is acceptable for calculating sample size for further studies. Assuming a dropout rate of 20%, we will plan to enroll a total of 96 participants with 48 in each group.

#### 2.2.2. Recruitment of Participants

Participants will be recruited from the four hospitals mentioned above in outpatient clinics. Posters will be used outside the acupuncture clinics. The posters will contain brief introductions about the inclusion/exclusion criteria, the free acupuncture treatments offered to eligible participants, and the contact information of the researchers.

#### 2.2.3. Inclusion Criteria

Participants who meet all the following requirements will be allowed for enrollment:

1. nulliparous women, aged between 18 and 30 years old, diagnosed with PD (according to the criteria of the Primary Dysmenorrhea Consensus Guideline [34]), and in Cold and Dampness Stagnation pattern (based on a revised Chinese national guideline [30]),
2. moderate to severe primary dysmenorrhea (PD) (≥40 mm on VAS-P) for three consecutive menstrual cycles,
3. duration of PD (self-reported pain) varying from 6 months to 15 years,
4. written informed consent.

#### 2.2.4. Exclusion Criteria

Exclusion criteria are as follows:

1. secondary dysmenorrhea (e.g., endometriosis and fibroids),
2. irregular/infrequent menstrual cycles (i.e., beyond the typical range of 21- to 35-day cycle),
3. complication with severe diseases (e.g., cerebral, liver, kidney, or hematopoietic system diseases), mental defects, or asthma,
4. pregnancy,
5. use of analgesic medication in 24 hours before treatment,
6. having a professional acupuncture background,
7. having potentially poor treatment compliance (e.g., unstable working and living situation or difficulty in following up).

### 2.3. Randomization and Blinding

**Figure 1:**
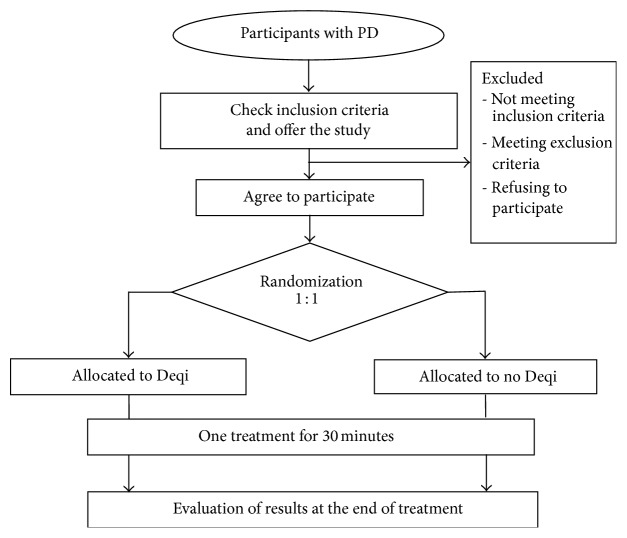
Flow diagram for the study. Work scheme with description of assessment visits and times.

The central randomization will be performed by the Center for Evidence-Based Chinese Medicine affiliated to Beijing University of Chinese Medicine in China, using complete randomization to generate the random allocation sequence. Once a participant is included, the researcher will contact the randomization center for the group allocation of the participant, and the acupuncturist will be informed by telephone. Participants will be allocated at a 1 : 1 ratio (Figure 1).

The participants will be informed that they will have a 50% chance of being allocated in either of the two acupuncture groups, and both groups will be potentially effective; hence, participants will be blinded to the group allocation. The patients will also be informed of the possible risks associated with acupuncture (hematoma or fainting). The acupuncturist cannot be blinded due to the specific nature of intervention. Outcome assessors and personnel who will deal with data collection and data analysis will be blinded throughout the entire trial.

### 2.4. Interventions

All participants, when their VAS-P score of menstrual pain is equal to or more than 40 mm on the first day of menstruation, will each receive an acupuncture treatment for 30 minutes at bilateral SP6 acupoints. The SP6 lies on the tibial aspect of the leg, is posterior to the medial border of the tibia, and is 3 B-cun (proportional bone cun) superior to the prominence of the medial malleolus [35].

*(a) Deqi Group*. The treatment will be performed after sterilizing the skin on the areas where the needles will be inserted, with participants lying face up. Using single-use sterile needles (Zhongyan Taihe, Wuxi Jiajian Medical Instrument Co. Ltd., Jiangsu, China) of 0.30 mm calibre and 40 mm length, a needle will be vertically inserted 1 cun in depth and will be manipulated by lifting-thrusting and twirling methods for 30 seconds to achieve Deqi.

*(b) No Deqi Group*. Single-use sterile needles (Zhongyan Taihe, Wuxi Jiajian Medical Instrument Co. Ltd., Jiangsu, China) of 0.18 mm calibre and 13 mm length will be used. The treatment will be performed after sterilizing the skin on the areas where the needles will be inserted, with participants lying face up. A needle will be vertically inserted 0.1 cun in depth. No manipulation will be performed after insertion of needles to avoid Deqi.

### 2.5. Outcome Measures

#### 2.5.1. Primary Outcome Measures

The changes of pain will be measured by visual analogue scale for pain (VAS-P), before and after the treatment. The validity and reliability of the VAS-P scale have been proven [36–38] and it was employed in our previous similar studies [29, 30]. The scale measures a continuous quantitative variable varying from 0 mm (no pain) to 100 mm (worst pain ever).

#### 2.5.2. Secondary Outcome Measures

1. The changes of the anxiety are measured by visual analogue scale for anxiety (VAS-A), before and after the treatment. This scale has been used in a PD study [39]. The scale measures a continuous quantitative variable varying from 0 mm (no anxiety) to 100 mm (almost death).
2. The changes of temperature at SP6 (Sanyinjiao) and CV4 (Guanyuan) acupoints (located at the lower abdomen, 3 B-cun inferior to the centre of the umbilicus, on the anterior median line [35], e.g., in the skin area corresponding to the uterus) are measured by a digital infrared thermographic imaging device (USA, FLIR Systems Inc., SC640) before and after treatment. It is demonstrated that acupuncture can affect skin temperature at the needling acupoint, and it is feasible to detect the changes of the skin temperature by using a thermographic camera at a certain acupoint during acupuncture treatment [40].
3. Adverse events: the possible side effects and adverse reactions during the treatment will be recorded in the case report form (CRF).

### 2.6. Data Collection

The data required for evaluating the influences of Deqi on acupuncture therapy will be collected at baseline and the completion of intervention. Data will be obtained via physical measurements. Data collection instruments and the study timeline are summarized in Table 1.

A case report form (CRF) has been designed, to include the variables of interest, which will be completed by the corresponding researcher at each research center. After the trial finished, the information obtained will be input to an electronic database, for subsequent statistical analysis.

### 2.7. Data Storage and Confidentiality

All CRFs will be stored in a locked cabinet at a study office in School of Acupuncture, Moxibustion and Tuina, Beijing University of Chinese Medicine, and will have a unique identification number. Data will be input to an electronic database, and the access to the database is restricted to the study team.

### 2.8. Statistical Analysis

All analyses will be performed using SPSS 17.0 (SPSS Inc., Chicago, IL). Potential differences across study groups on demographic and clinical history variables will be compared by means of analysis of covariance (ANCOVA). Repeated-measures analysis of variance (ANOVA) will be utilized for the analysis of VAS-P and VAS-A scores and temperatures at SP6 (Sanyinjiao) as well as CV4 (Guanyuan) acupoint. *P* < 0.05 will be denoted as significant.

Although most participants of the Deqi group will experience Deqi, there will probably be some exceptions that participants in the group will not experience Deqi. Therefore, we will redivide all participants into the real Deqi group and unreal Deqi group according to a self-designed Acupuncture Deqi Clinical Assessment Scale (ADCAS) grade after treatment. We will perform a secondary analysis with similar statistical method to explore whether the real Deqi has influences on the effect of acupuncture. We will compare the first and second analysis results.

## 3. Discussion

The trial is designed to illustrate Deqi effect on acupuncture treatment. It will estimate Deqi state of solo acupoint and compare analgesic right after needling. All participants will be randomly divided into Deqi or no Deqi group. Unlike other impact factors of acupuncture treatment, Deqi is a group of specific subjective sensations which can only be felt during acupuncture by both patients and acupuncturists. It is impossible to randomly allocate participants into a Deqi or no Deqi group, so there have been no Deqi randomized controlled trials (RCTs). However, whether Deqi can be achieved during acupuncture can be predicted to a certain extent; therefore, we will randomly divide all participants into Deqi or no Deqi group. We also employ manipulation technique to promote Deqi in the Deqi group, while in the no Deqi group we avoid Deqi according to TCM theory and experts' experience.

In this study, we will use an important self-designed instrument—Acupuncture Deqi Clinical Assessment Scale (ADCAS). The scale shows the intensity of participants' sensation, ranging from 0 to 4: 0 (no), 1 (slight), 2 (mild), 3 (obvious), and 4 (strong). It will be firstly used as an instrument for redividing all the included participants in reanalysis. Actually, some researchers and their colleagues have attempted to qualify and quantify sensations of Deqi [41–47] and designed several Deqi scales or questionnaires like the Vincent scale, the Park questionnaire, the MGH Acupuncture Sensation Scale (MASS), and Southampton Needling Sensation Questionnaire (SNSQ). Some of the scales or questionnaires have been used in Deqi trials [48–50] and MASS even has a Chinese edition [51]. However, though Deqi sensation can be felt by patients and acupuncturists, all the current scales take no consideration of the Deqi sensation felt by acupuncturists' hands, which decreases the reliability of those scales.

Additionally, all the previous scales or questionnaires derived from a pain questionnaire—McGill Pain Questionnaire (MPQ) [52], which are not based on TCM theory. Thus, we designed this new Deqi questionnaire based on the previous instruments and especially followed TCM theory and rigorous methodology, such as selecting the items included in the Deqi questionnaire and testing reliability and validity. In fact, the reliability and validity of ADCAS were evaluated by a study with 73 PD patients needled on SP6, in which the disease and selected acupoint were the same as in this study, and it had a good result (the paper will be published later).

As a subjective sensation, Deqi has no objective measure instrument by far. This trial will explore the possibility of acupoint temperature changing as a sign of Deqi and will be devoted to the provision of a tool to determine Deqi.

This trial, in accordance with the STRICTA [53] and good clinical practice guidelines (GCP), will be helpful in explaining the role of Deqi in acupuncture analgesia, provide new objective evidence for Deqi, and improve the understanding of complicated mechanisms of acupuncture in practice.

## Figures and Tables

**Table 1:** Data collection at different assessment points. Example of outcome measurements.

Variable	T0	T1
VAS-P	X	X
VAS-A	X	X
ADCAS		X
Temperature at SP6, CV4	X	X
Side effects and adverse reactions		X
Sociodemographic data	X	

Note: VAS-P: visual analogue scale for pain (0–100); VAS-A: visual analogue scale for anxiety (0–100); ADCAS: Acupuncture Deqi Clinical Assessment Scale; SP6: Sanyinjiao; CV4: Guanyuan; T0: before acupuncture treatment; T1: after acupuncture treatment.
